# Relationships between elementary school children's experiences in helping older adults and their attitudes toward individuals with dementia and intention to help: A cross‐sectional survey aiming to develop age‐friendly education

**DOI:** 10.1111/ggi.70014

**Published:** 2025-02-26

**Authors:** Ayumi Igarashi, Manami Takaoka, Hiroshige Matsumoto, Noriko Yamamoto‐Mitani

**Affiliations:** ^1^ Department of Gerontological Homecare and Long‐Term Care Nursing, Division of Health Science and Nursing, Graduate School of Medicine The University of Tokyo Tokyo Japan; ^2^ Department of Community Health Nursing/Public Health Nursing, Division of Health Science and Nursing, Graduate School of Medicine The University of Tokyo Tokyo Japan

**Keywords:** age‐friendly education, attitudes toward individuals with dementia, community‐based integrated care system, elementary school children, helping behaviors

## Abstract

A survey of 116 sixth‐grade students found that 39% had supported older adults in the past year. Those with prior experience showed significantly higher attitudes and helping intentions toward people with dementia. Encouraging practical helping experiences may foster awareness and willingness to assist older adults in the community.

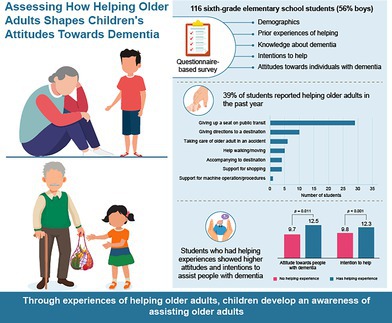

Dear Editor,

Awareness of aging and dementia among children is crucial for building a community‐based symbiotic society. To cultivate knowledge and encourage helping behaviors toward individuals with dementia, the Japanese government has promoted the “Dementia Supporter Training Program” for primary and junior high school students.^1^ Additionally, the “Basic Law on Dementia to Promote the Realization of a Symbiotic Society” underscores the importance of integrating dementia education into school curricula across Japan.^2^ While these efforts emphasize the need for dementia education, the specific strategies that effectively foster the intention to engage in helping behaviors remain unclear. There is limited empirical evidence on whether actual experiences of helping older adults are associated with children's intentions to engage in such behaviors. To address this gap, this study examined the relationships between elementary students' experiences of helping older adults and their knowledge, attitudes, and intentions toward individuals with dementia. Our aim was to provide foundational insights for educators and program developers in designing age‐ and dementia‐friendly education programs.

A questionnaire survey, including 116 sixth‐grade students from two elementary schools in Hinode, Tokyo, was conducted from June to October 2023. All the students responded to the survey, which was conducted by each elementary school as part of classroom evaluations. The schools removed personal information from the response data to facilitate anonymization before providing it to the researchers for secondary use. The data collected included demographic information, past experiences of helping unfamiliar older adults encountered while outside in the past year (“helping behaviors”),^3^ knowledge about dementia (“knowledge,” 6 items), attitudes toward individuals with dementia (“attitudes,” 14 items),^4^ and intention to engage in helping behaviors (“intention to help,” 4 items).^5^ Knowledge was assessed based on the number of correct answers (α = 0.620). Attitudes were scored based on the following responses, with their corresponding points (α = 0.813): “agree” (2 points), “somewhat agree” (1 point), “disagree/somewhat disagree” and “don't know” (0 points). For the intention to help, the total score was calculated using a four‐point scale ranging from “agree” (4 points) to “disagree” (1 point) (α = 0.800). After descriptive statistics were calculated, differences in knowledge, attitudes, and intention to help were analyzed using *t*‐tests between students with and without experience in helping older adults. This study was approved by the Research Ethics Committee of the Graduate School of Medicine at the University of Tokyo.

Among the 116 children, 56.0% were male, 12.9% lived with their grandparents, 25.9% had family member(s) working in healthcare settings, and 6.9% had family member(s) with dementia (Table 1). The mean scores (±standard deviation) for knowledge, attitudes, and intention to help were 2.8 (±1.6), 10.7 (±5.8), and 10.7 (±3.2), respectively. Forty‐five children (38.8%) reported assisting an older adult in the previous year (Table 1). Students with prior helping experiences scored significantly higher in attitudes (12.5 vs. 9.7, *P* = 0.011) and intentions to help (12.3 vs. 9.8, *P* < 0.001) than did those without. However, no significant difference in knowledge was observed between the two groups (2.7 vs. 2.8, *P* = 0.738; Table 1). Girls showed significantly higher intentions to help than boys (11.8 vs. 10.0, *P* = 0.004; data not shown in the table). No significant associations were found between knowledge, attitudes, or intentions and factors such as school, living with grandparents, or having family members in care‐related work or with dementia.

**Table 1:** Characteristics of the participants and the association with the experience of helping behaviors (*n* = 116)

	All (*n* = 116)		Having no helping experience (*n* = 71)		Having helping experience* (*n* = 45)		*P*‐value***
	*n*	(%)	*n*	(%)	*n*	(%)	
	Mean	±SD	Mean	±SD	Mean	±SD	
School							
School A	63	(45.7)	32	(45.1)	21	(46.7)	0.866
School B	53	(54.3)	39	(54.9)	24	(53.3)	
Sex							
Boy	65	(56.0)	45	(64.3)	20	(44.4)	0.128
Girl	42	(36.2)	21	(29.6)	21	(46.7)	
No answer	9	(7.8)	5	(7.0)	4	(8.9)	
Living with grandparent(s)	15	(12.9)	10	(14.1)	5	(11.1)	0.642
Having family member(s) working in a hospital/nursing home	30	(25.9)	17	(23.9)	13	(28.9)	0.553
Having family member(s) with dementia	8	(6.9)	4	(5.6)	4	(8.9)	0.500
Knowledge of dementia	2.8	±1.6	2.8	±1.6	2.7	±1.6	0.738
Attitude toward persons with dementia (*n* = 115)	10.7	±5.8	9.7	±5.8	12.5	±5.6	0.011
Intention of helping behaviors (*n* = 112)	10.7	±3.2	9.8	±3.2	12.3	±2.4	<0.001
Experiencing helping behavior(s) in the past year	45	(38.8)					
Giving up a seat on public transport	29	(64.4)**					
Giving directions to a destination	10	(22.2)**					
Help walking/moving	5	(11.1)**					
Taking care of older adult in an accident	6	(13.3)**					
Accompanying to destination	4	(8.9)**					
Support for shopping	4	(8.9)**					
Support for machine operation/procedures	1	(2.2)**					

SD, standard deviation.

* Students who reported engaging in at least one helping behavior were categorized as “having helping behavior.”

** Percentage of 45 participants with experience in helping as denominator.

*** Categorical variables were analyzed using the chi‐square test, and continuous variables were analyzed using the *t*‐test.

Our findings showed that elementary students' helping behaviors toward older adults ranged from acts that can be taken immediately on the spot, such as yielding seats or giving directions, to more advanced helping behaviors that require a step forward, such as taking care of older adults in an accident or in poor health, or aiding them in walking and carrying baggage.^3^ In our study, students helping older adults demonstrated a more positive attitude and a greater willingness to provide help to individuals with dementia, whereas previous studies have assumed that positive attitudes toward those with dementia and helping intentions result in helping behavior.^5^ The findings of both previous studies and our study suggest the possibility of a bidirectional influence. Our study suggests that the experience of helping older adults may reduce children's prejudice and foster a culture of helping older adults, including individuals with dementia.

To the best of our knowledge, this is the first study to examine the relationships among elementary students' attitudes toward individuals with dementia, their intentions to help, and their actual experiences in helping older adults. Future research should focus on developing age‐ and dementia‐friendly education programs that incorporate opportunities for elementary school students to engage in helping behaviors to foster positive attitudes toward older adults and facilitate competency in caring for them, ultimately contributing to a more symbiotic society.

## Disclosure statement

The authors declare that they have no conflicts of interest.

## Data Availability

Research data are not shared.
